# 

**Published:** 1892-06-11

**Authors:** 


### SPECIAL HOSPITAL SUNDAY NUMBER.

CONTENTS.

**Faith and Science at the Hospitals** - 161

**Passing Topics.**—"Must" - 162

**The Doctor's Armchair.**—Anæsthetics - 163

**Poisons and Poisoners**—The Marquise de Brinvilliers - 164

**Medical History from the Earliest Times.** By E. T. WITHINGTON, M.A., M.B.Oxon. XII.—The Successors of Hippocrates—The Dogmatic School - 165

**Everybody's Page** - 166

**Annotations.**—Mirth and Medicine—Scarlet Fever Treatment and Disinfection—Town Children's Summer - 167

**Notes and News** - 168

**The Work of the Hospitals.** I.—Hospital Accommodation for Bread-winners. II.—Hospital Accommodation for Women. III.—What the Hospitals do for the Middle Classes. IV.—With the Children. V.—The Chronic Diseases of Children; Incurable Homes; and other Supplementary Work - 169

**Editor's Letter Box.**—Infectious Diseases in Private Houses - 176

**EXTRA SUPPLEMENT.—THE NURSING MIRROR.**

**En Passant.**—Queen Charlotte's Lying-in Hospital—Redruth—Worcester General Infirmary—Nurses' Wages—Advance Guardians!—Tea and Talk—Middlesex Hospital—Wrentham Cottage Training Home—Work and Health—A Warning Note lxxiii

**Ventilation, Disinfection, and Diet.** By P. CALDWELL SMITH, M.D. IX.—Disinfection (*continued*) - lxxiv

**Asylum Notes—Presentations** - lxxv

**For Reading to the Sick.**—The Shorn Lamb - lxxv

**Proposed Midwives Bill** - lxxvi

**Notes and Queries** - lxxvi

**Wants and Workers** - lxxvi

**Everybody's Opinion.**—The Needless Dread of Nursing Smallpox Cases—Private Nurses and their Ways—St. Olave's Infirmary, Rotherhithe - lxxvii

**The Nurses' Bookshelf.**—Nursing and Hygiene - lxxvii

**Four Months in a Hospital Ward.** IV. - lxxviii

